# Analysis of influencing factors of orthopedic nurses' spiritual care competencies based on structural equation model

**DOI:** 10.3389/fpubh.2024.1462724

**Published:** 2024-11-13

**Authors:** Xiaoju Chen, Renzhi Yuan, Yibing Du, Aihong Fan

**Affiliations:** ^1^Department of Spine Surgery, Hefei First People's Hospital, Hefei, China; ^2^Department of Joint Surgery, Hefei First People's Hospital, Hefei, China; ^3^Department of Nursing, Hefei First People's Hospital, Hefei, China

**Keywords:** spiritual care, orthopedic nurse, professional identity, hospital ethical climate, structural equation model

## Abstract

**Purpose:**

To comprehensively understand the spiritual care competencies of orthopedic nurses and analyze the factors that affect them.

**Methods:**

This study employed convenience sampling to conduct a cross-sectional survey among orthopedic nurses at Hefei First People's Hospital. Data was collected utilizing a general information questionnaire, alongside the Chinese versions of the Spiritual Care Competence Scale, the Spiritual Care Perspective Scale, the Professional Identity Assessment Scale, and the Hospital Ethical Climate Scale. Guided by the Person-Environment Fit theory and the Triadic Reciprocal Determinism theory, a structural equation model was utilized to analyze the influencing factors and pathways related to the spiritual care competence of orthopedic nurses.

**Results:**

A total of 112 valid questionnaires were obtained, yielding an overall score of 68.92 ± 11.03 for orthopedic nurses' spiritual care competencies, with an average score of 3.14 ± 0.50 per item. The results of the Pearson correlation analysis revealed a significant positive correlation (all *P* < 0.01) between spiritual care competence and the scores for spiritual care perspective, professional identity assessment, and hospital ethical climate. The spiritual care perspective, professional identity, and hospital ethical climate emerged as the influencing factors of orthopedic nurses' spiritual care competence, exhibiting total effects of 0.30, 0.53, and 0.85, respectively. Notably, the hospital ethical climate exerts an indirect influence on spiritual care competence through the mediation of spiritual care perspective and professional identity, with the indirect effect accounting for 61.18% of the total effect.

**Conclusion:**

There remains significant potential for enhancing the spiritual care competencies of orthopedic nurses. The hospital's ethical climate not only has a direct and positive impact on spiritual care competence but also indirectly influences it through the lens of nurses' spiritual care perspectives and professional identity assessments. Hospital administrators may wish to consider strategies for bolstering the hospital's ethical climate, fostering a deeper spiritual care perspective and heightened professional identity among nurses, and ultimately improving their spiritual care competence.

## 1 Introduction

In 1998, the World Health Organization introduced the concept of spiritual health as the fourth dimension of health (1). Spiritual care entails a mode of care where medical personnel, after identifying patients' spiritual needs, assist them in seeking the meaning of life and achieving peace and comfort through listening, companionship, respect, and various other means (2, 3). For patients, spiritual needs are universal, making spiritual care a crucial component of holistic care (4, 5). Studies have demonstrated that spiritual care can alleviate patient pain, enhance overall quality of life, and help overcome fears related to disease and surgery (4, 6, 7). There is a correlation between caregivers' spiritual care competence, their spiritual health, and their performance in addressing patients' spiritual needs (8). Pain is a prevalent symptom among orthopedic patients, as trauma, surgery, postoperative fixation, and functional exercise can all induce varying levels of discomfort, adversely affecting their physiological and psychological wellbeing (9). Given this, providing spiritual care to orthopedic patients is imperative; however, research on the spiritual care competency of orthopedic nurses remains scarce. To address this gap, the present study was conducted. The Person-Environment Fit theory (10) and the Triadic Reciprocal Determinism theory (11) serve as frameworks to guide the understanding of factors influencing spiritual care competency. These theories highlight two primary aspects of spiritual care competency among nursing staff: the work environment and subjective factors of organizational members (cognition, concepts, emotions, etc.). Regarding the work environment, the hospital ethical climate, which encompasses the climate, attitude, and ethical judgments perceived by medical staff, is a vital component. It can enhance their professional behavior (12, 13) and promote a spiritual care perspective among medical staff (14). Subjective factors are primarily reflected in nurses' cognition, concepts, and emotional experiences concerning nursing work and spiritual care. Professional identity, a subjective amalgamation of beliefs, judgments, and attitudes toward oneself and one's professional role, encompasses professional self-evaluation, self-experience, and self-control (15–17). It serves as an internal motivating factor in nurses' career development and a pivotal psychological foundation for determining their professional behavior tendencies (18, 19). Individuals with a higher professional identity demonstrate a stronger ability to empathize with others, thereby providing better spiritual care to patients. The subjective experience of spiritual care, known as the spiritual care perspective, refers to the spiritual values upheld by nurses in clinical practice, including their understanding of spirituality, spiritual health, and spiritual care (20). It can be categorized into a cognitive perspective and an implementation perspective (21). The spiritual care perspective is crucial for the smooth implementation of clinical spiritual care practices and influences an individual's spiritual care competence to some extent. Professional identity and the hospital ethical atmosphere may directly or indirectly impact nurses' spiritual care competencies. Therefore, this study constructed a structural equation model based on the Person-Environment Fit theory and the Triadic Reciprocal Determinism framework, exploring the mechanisms through which the spiritual care perspective, professional identity, and hospital ethical atmosphere influence nurses' spiritual care competencies. This provides a theoretical foundation for enhancing nurses' spiritual care competencies.

## 2 Methods and analysis

### 2.1 Participants

From April to May 2024, a questionnaire survey was conducted among orthopedic nurses at Hefei First People's Hospital, utilizing the convenience sampling method. The inclusion criteria were as follows: (1) registered nurses with more than 1 year of experience in orthopedic nursing; (2) individuals who volunteered to participate in the study. The exclusion criteria included: (1) nurses pursuing advanced studies; (2) nurses who were away for advanced studies; (3) nurses on vacation during the period of investigation.

### 2.2 Calculation of sample size

The recommended sample size for structural equation modeling should be at least 10 times the number of included variables. In this study, the structural equation model incorporated four variables: spiritual care competencies, spiritual care perspective, professional identity assessment, and hospital ethical climate. Taking into account a potential 15% invalid questionnaires, the minimum required sample size was calculated to exceed 46 cases. Ultimately, the final sample size for this study amounted to 112 cases, comfortably meeting the specified requirements.

### 2.3 Survey instruments

#### 2.3.1 General information questionnaire

The content, designed by the researchers themselves, encompasses gender, age, religious beliefs, educational level, professional title, duration of work in orthopedics, marital status, and prior participation in spiritual care education programs.

#### 2.3.2 Chinese versions of the spiritual care competence scale

Wei et al. (22) introduced this scale into Chinese, which comprises six dimensions: assessment and implementation of spiritual care, professionalization and quality improvement of spiritual care, personal support and patient counseling, referral to professionals, attitude toward the patient's spirituality, and communication. The scale consists of a total of 22 items. Utilizing the Likert 5-level scoring system, responses are scored as follows: “never” = 1 point, “rarely” = 2 points, “sometimes” = 3 points, “often” = 4 points, and “always” = 5 points, yielding a total possible score range of 22–110 points. A higher score indicates a stronger spiritual care ability. The Cronbach's alpha coefficient for the scale is 0.974.

#### 2.3.3 Chinese versions of the spiritual care perspective scale

This scale was developed by Taylor et al. (21) and subsequently translated into Chinese by Liu et al. (23). It encompasses two dimensions: cognitive perspective and implementation perspective, comprising a total of 10 items. Using the Likert 5-point scoring system, responses are scored as follows: “strongly agree” = 1 point, “agree” = 2 points, “uncertain” = 3 points, “disagree” = 4 points, and “strongly disagree” = 5 points, resulting in a total possible score range of 10–50 points. A higher score indicates a more positive attitude toward providing spiritual care among nurses. The Cronbach's alpha coefficient for the scale is 0.872.

#### 2.3.4 Professional identity assessment scale

This scale, developed by Liu et al. (24), comprises five dimensions with a total of 30 items. These dimensions include professional cognitive evaluation, professional social skills, professional social support, handling professional setbacks, and professional self-reflection. Utilizing the Likert 5-point scoring system, responses are scored as follows: “strongly agree” = 1 point, “agree” = 2 points, “uncertain” = 3 points, “disagree” = 4 points, and “strongly disagree” = 5 points, yielding a total possible score range of 30–150 points. A higher score indicates a higher level of professional identity among nurses. The Cronbach's alpha coefficient for this scale is 0.931.

#### 2.3.5 Hospital ethical climate survey

This scale was developed by Professor Olson (25) and later revised for use in Chinese by Wang (26). It encompasses five dimensions and consists of 26 items, specifically addressing the nurse's relationship with nurses, doctors, patients, hospitals, and managers. Using the Likert 5-point scoring system, responses are scored as follows: “strongly agree” = 1 point, “agree” = 2 points, “uncertain” = 3 points, “disagree” = 4 points, and “strongly disagree” = 5 points, resulting in a total possible score range of 26–130 points. A higher total score indicates a more favorable perception of the hospital's ethical climate by the nurse. The Cronbach's alpha coefficient for this scale is 0.915.

### 2.4 Survey methods

The researcher contacted the relevant departments of the hospital and obtained the consent of the department leaders. Before the formal start of the survey, a survey team was formed, and training was provided on the guidance language, informed consent, and anonymity principles prior to the distribution of the questionnaire. For information collection, electronic questionnaire filling tools were utilized; nurses only needed to scan the questionnaire QR code on their mobile phones to complete the questionnaire. The content of the questionnaire did not involve the nurse's name, job number, or other identifying information, thus ensuring the anonymity of the information. In the design of the electronic questionnaires, measures such as setting a minimum filling time and requiring all questions to be answered before submission were implemented to ensure the availability and completeness of the information. The research team members, who had undergone unified training, ventured into the ward and employed unified guidance language to elucidate the questionnaire filling requirements. After obtaining the informed consent of the nurses, the survey was conducted anonymously. The participants responded to the questions on the spot and submitted their answers immediately.

### 2.5 Statistical analysis

Data analysis was conducted using SPSS 22.0 and Amos 28.0 software. Quantitative data were described using the mean and standard deviation, denoted as (X ± s), while count data were described in terms of frequency and percentage. Pearson correlation analysis was employed to assess the correlation between spiritual care perspective, hospital ethical climate, professional identity, and spiritual care competence. A structural equation model was constructed using Amos 28.0 software, with model parameters estimated using the Maximum Likelihood method. The initial model was refined using the Modified Index and Model Fit Index. The Bootstrap method was utilized to perform 5,000 repetitions of sampling to analyze the mediating effect. A *p*-value of < 0.05 was considered statistically significant.

## 3 Results

### 3.1 Analysis of the spiritual care competencies of orthopedic nurses

#### 3.1.1 Score of spiritual care competencies of orthopedic nurses

The total score for the spiritual care competencies of orthopedic nurses is (68.98 ± 11.03), with an overall average score of (3.14 ± 0.50). Among the items, “Attitude toward the patient's spirituality” received the highest average score of (3.30 ± 0.63), while “Referral to professionals” scored the lowest, with an average of (2.99 ± 0.61). The detailed breakdown is presented in Table 1.

**Table 1:** Score of spiritual care competencies of orthopedic nurses.

**Item**	**Score ($\bar{X}$ ±s)**	**Sort**
Assessment and implementation of spiritual care	3.11 ± 0.63	3
Professionalization and improving the quality of spiritual care	3.04 ± 0.59	5
Personal support and patient counseling	3.11 ± 0.58	4
Referral to professionals	2.99 ± 0.61	6
Attitude toward the patient's spirituality	3.30 ± 0.63	1
Communication	3.29 ± 0.61	2
Total average score	3.14 ± 0.50	
**Total score**	68.98 ± 11.03	

#### 3.1.2 Comparison of total average scores of spiritual care competencies among orthopedic nurses with different characteristics

The results indicated a statistically significant difference in the total average scores of spiritual care competencies among orthopedic nurses based on their religious beliefs, educational level, duration of orthopedic work experience, and prior participation in spiritual care education programs (*P* < 0.05). These findings are summarized in Table 2.

**Table 2:** Comparison of total average scores of spiritual care competencies among orthopedic nurses with different characteristics.

**Item**	**Number**	**Score**	***F*/*t*-value**	***P***
**Gender**			−1.13	0.261
Man	7	2.92 ± 0.40		
Woman	105	3.15 ± 0.51		
**Religious beliefs**			8.662	0.0001
Yes	8	3.80 ± 0.48		
No	104	3.08 ± 0.48		
**Educational level**			13.19	0.0001
Secondary vocational school diploma	19	2.76 ± 0.45		
Three-year college diploma	43	3.05 ± 0.57		
Bachelor degree or above	50	3.36 ± 0.33		
**Professional title**			1.312	0.274
Nurse	42	3.25 ± 0.45		
Senior nurse	24	3.09 ± 0.50		
Supervisor nurse	36	3.07 ± 0.53		
Co-chief nurse or above	10	3.00 ± 0.58		
**Length of orthopedic work**			4.841	0.003
1–5 years	20	3.47 ± 0.28		
6–10 years	43	3.04 ± 0.49		
11–15 years	29	3.17 ± 0.47		
16 years or above	20	2.95 ± 0.60		
**Marital status**			−1.869	0.068
Having a spouse	90	3.10 ± 0.53		
No spouse	22	3.27 ± 0.34		
**Previous participation in spiritual care education programs**			16.38	0.0001
Yes	37	3.68 ± 0.15		
No	75	2.86 ± 0.38		

*P* < 0.05 indicates a statistically significant difference.

### 3.2 Score of spiritual care perspective, professional identity assessment, and hospital ethical climate

The results revealed that the score for the Spiritual Care Perspective was (29.54 ± 3.03), the score for the Professional Identity Assessment was (89.67 ± 7.97), and the score for the Hospital Ethical Climate was (96.38 ± 13.51). The scores for each respective dimension are presented in Table 3.

**Table 3:** Orthopedic nurses' spiritual care perception, professional identity assessment, and hospital ethical climate scores (*n* = 112).

**Item**	**Score (X̄ ±s)**	**Average score of entries (X̄ ±s)**
**Spiritual care perception**	29.54 ± 3.03	2.95 ± 0.85
Cognitive perspective	20.87 ± 2.82	2.98 ± 0.84
Implement perspective	8.67 ± 1.12	2.89 ± 0.86
**Professional identity assessment**	89.67 ± 7.97	3.21 ± 0.89
Professional cognitive evaluation	12.17 ± 1.80	2.96 ± 0.86
Professional social skills	15.96 ± 1.35	3.04 ± 0.91
Professional social support	17.55 ± 2.40	3.41 ± 0.88
Dealing with professional setbacks	21.09 ± 2.48	3.40 ± 0.86
Professional self reflection	21.90 ± 2.31	3.54 ± 0.78
**Hospital ethical climate**	96.38 ± 13.51	3.45 ± 0.88
Relationship with nurses	26.63 ± 4.46	3.29 ± 0.80
Relationship with patients	18.24 ± 3.75	3.99 ± 0.61
Relationship with doctors	20.46 ± 2.79	2.93 ± 0.98
Relationship with managers	20.42 ± 3.10	3.51 ± 0.82
Relationship with the hospital	10.64 ± 1.41	3.65 ± 0.71

### 3.3 Correlation analysis between the spiritual care competencies of orthopedic nurses and their spiritual care perspective, professional identity assessment, and hospital ethical climate

The results of the Pearson correlation analysis demonstrated a positive correlation between spiritual care competencies and spiritual care perspective (*r* = 0.577), professional identity assessment (*r* = 0.914), and hospital ethical climate (*r* = 0.879), with all correlations being statistically significant at *P* < 0.01.

### 3.4 Construction of a structural equation model for factors influencing the spiritual care competencies of orthopedic nurses

#### 3.4.1 Construction of a structural equation model

**Figure 1:**
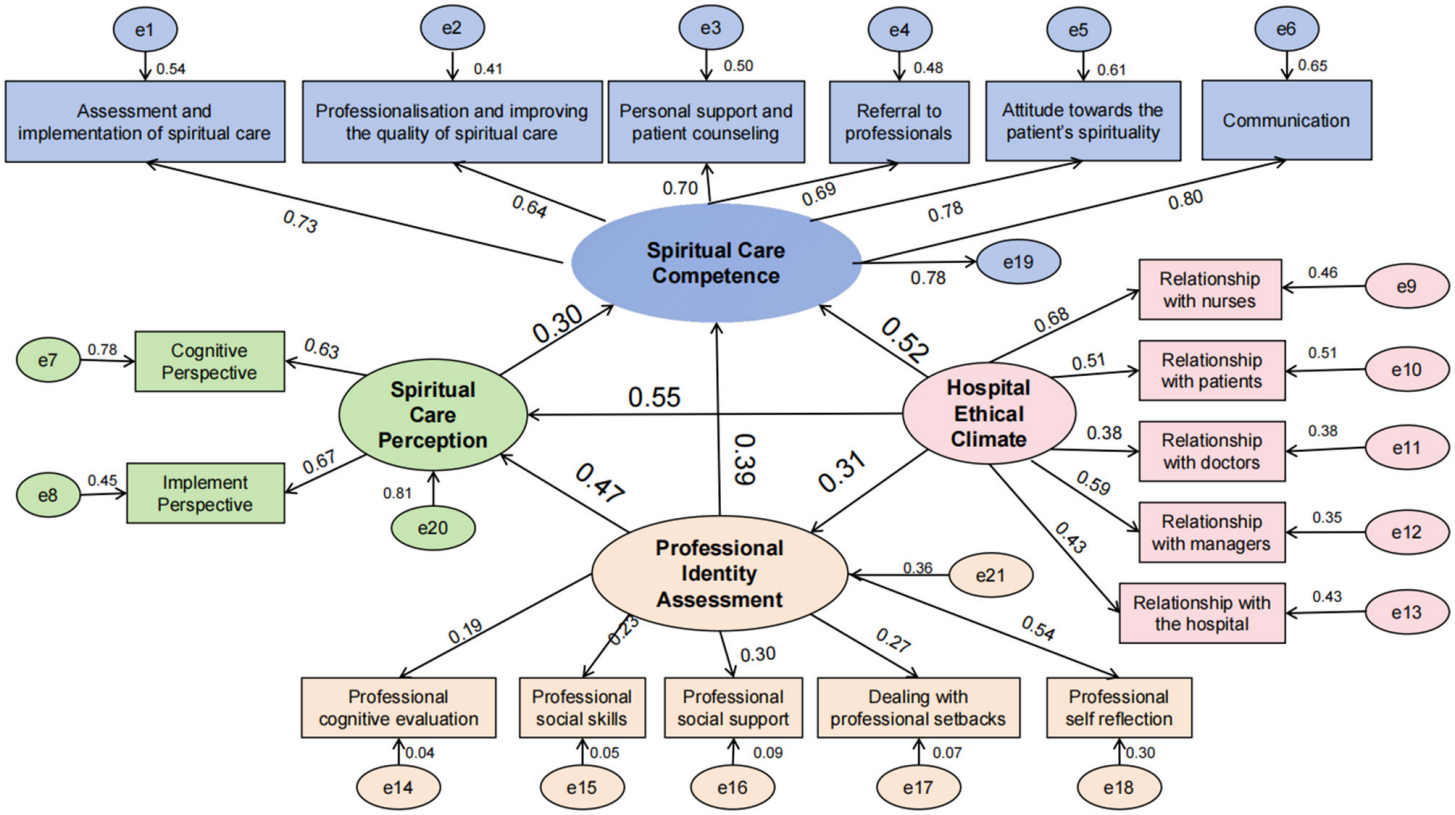
Structural equation model of factors influencing the spiritual care competences of orthopedic nurses.

Based on the Person-Environment Fit theory (10) and the Triadic Reciprocal Determinism theory (11), a structural equation model was constructed to investigate the influencing factors of orthopedic nurses' spiritual care competencies. In this model, the hospital ethical climate served as the independent variable, while Spiritual Care Perspective and professional identity assessment acted as mediating variables, and spiritual care competencies were the dependent variable. As illustrated in Figure 1, all fitting indicators of the model met the required standards, suggesting that the paths are valid. Specifically, the Chi-Square/degree of freedom ratio (χ2/df) was 2.526, the Root-Mean-Square-Error of Approximation (RMSEA) was 0.047, the Goodness-of-Fit Index (GFI) was 0.940, the Normed Fit Index (NFI) was 0.978, and the Comparative Fit Index (CFI) was 0.967.

#### 3.4.2 Path results

The hospital ethical climate has a direct positive impact on spiritual care competencies (β = 0.52) and also exerts indirect effects through three pathways: hospital ethical climate → professional identity assessment → Spiritual Care Perspective → spiritual care competencies (β = 0.31 × 0.47 × 0.30 = 0.04), hospital ethical climate → Spiritual Care Perspective → spiritual care competencies (β = 0.55 × 0.30 = 0.17), and hospital ethical climate → professional identity assessment → spiritual care competencies (β = 0.31 × 0.39 = 0.12). These indirect effects collectively account for 61.18% of the total effect. Additionally, the professional identity assessment has a direct positive impact on spiritual care competencies (β = 0.39) and an indirect effect mediated by the Spiritual Care Perspective (β = 0.47 × 0.30 = 0.14). Furthermore, the Spiritual Care Perspective has a direct positive impact on spiritual care competencies (β = 0.30). The effect decomposition of each variable is detailed in Table 4.

**Table 4:** Effect decomposition of factors influencing the spiritual care competence of orthopedic nurses.

**Model path**	**Indirect effect**	**Direct effect**	**Total effect**
Hospital ethical climate → spiritual care competencies	0.33	0.52	0.85
Professional identity assessment → spiritual care competencies	0.14	0.39	0.53
Spiritual care perception → spiritual care competencies	/	0.30	0.30

## 4 Discussions

### 4.1 Analysis on the current situation of spiritual care competencies of orthopedic nurses

The results of this survey reveal that the overall average score for the spiritual care abilities of orthopedic nurses is (3.14 ± 0.50), with a total score of (68.98 ± 11.03), aligning with the findings of Li et al. (27). Currently, spiritual care in China is in its developmental phase, and its implementation in clinical practice and education (including institutional and continuing education) remains exploratory (28, 29). Despite patients experiencing spiritual distress, many nurses are unable to provide timely and effective spiritual care due to a lack of experience and skills, leading to their spiritual care competencies not being prominent (30). The five items with the lowest scores highlight that orthopedic nurses' practical abilities in spiritual care are still insufficient, impeding their capacity to address mental health issues of patients and their families in a timely and effective manner. There is a notable absence of spiritual care plans within overall care. Among all dimensions, the lowest score was observed in “Referral to professionals,” which may be related to the lack of referral awareness among nurses. This also potentially reflects that the hospital's ethical atmosphere and personal subjective factors can enhance nurses' spiritual care competence by improving their awareness of spiritual care. Secondly, even though nurses are aware that patients need referral, there is currently a lack of standardized spiritual care procedures and specific referral methods and processes in China (31). This underscores the need for researchers to explore suitable spiritual care models and procedures tailored to China, while clarifying the processes and personnel responsibilities involved. Nurses, as the medical professionals with the most frequent contact with patients, play a crucial role in promptly referring patients to ensure they receive appropriate spiritual care. Therefore, it is imperative to enhance nurses' referral capabilities. The top five scoring items indicate that most orthopedic nurses respect or accept patients' spiritual beliefs, reflecting commendable humanistic care. Notably, the attitude toward patients' spirituality scored highest, potentially due to the heightened emphasis on humanistic and holistic care in nursing, which fosters nurses' respect for patients' personalities, lives, and beliefs (32, 33).

### 4.2 The spiritual care perspective has a positive predictive effect on spiritual care competence

This study demonstrates that a more advanced spiritual care perspective positively influences nurses' ability to provide spiritual care, exhibiting higher competence. As the primary providers of spiritual care, nurses' spiritual perspective can directly shape their judgments and behaviors when offering spiritual support to patients (34, 35). Mthembu et al. (36) found in their research that nurses with adequate theoretical awareness are better equipped to provide spiritual care to patients. Therefore, managers should organize regular professional lectures on spiritual care to ensure that nurses have a clear understanding of its relevant content. By guiding them to consciously incorporate spiritual care into their work and integrate it into overall nursing, managers can enhance nurses' spiritual care competence.

### 4.3 Professional identity assessment has direct and indirect effects on spiritual care competence

① This study reveals that professional identity assessment has a direct impact on spiritual care competence. Individuals possessing high levels of professional identity hold a more favorable view of their profession, and this positive sentiment can empower them to surmount workplace challenges and exhibit a heightened level of subjective initiative in their duties, ultimately fostering the enhancement of nurses' spiritual care competence (37). ② Furthermore, the study uncovered that nurse professional identity assessment indirectly influences spiritual care competence through the lens of spiritual care perspective. Nurses with stronger professional identities harbor deeper convictions, judgments, and attitudes concerning their professional roles, which aids them in developing a richer comprehension of spiritual care perspective and enhancing their spiritual care competence (38). Consequently, the influence of professional identity assessment on nurses' spiritual care competence can be facilitated by bolstering their spiritual care perspective. In future endeavors, regular training sessions will be organized for nursing staff, inviting experienced and capable nurses to deliver lectures on professional identity. These sessions aim to inspire nurses to cultivate a stronger sense of professional identity with their nursing work, thereby enhancing their spiritual care competence.

### 4.4 The hospital ethical climate can directly affect spiritual care competence, or indirectly affect spiritual care competence through spiritual care perspectives and professional identity assessments

① The hospital ethical climate exerts a positive influence on spiritual care competence, suggesting that as the ethical climate in hospitals improves, so does the spiritual care competence of nurses. The hospital ethical climate pertains to the ethical atmosphere, attitudes, and judgments perceived by medical staff (39). According to the Person-Environment Fit theory, the environment in which organizational members operate can shape their professional behavior (40). Reflecting the ethical dimensions of the hospital work environment, the ethical climate influences nurses' comprehension and resolution of ethical dilemmas in clinical practice (41). A favorable hospital ethical climate steers nurses toward adopting appropriate attitudes and behaviors toward related nursing matters, thereby augmenting their spiritual care competence. ② The hospital ethical climate can indirectly influence spiritual care competence through the lens of the spiritual care perspective. It impacts medical staff's ethical stance toward related issues (42). Nurses, influenced by the hospital ethical climate, develop their own thoughts and perspectives on ethical matters, facilitating their understanding of the content and concepts associated with spiritual care. From both cognitive and practical perspectives, they cultivate a more accurate spiritual care perspective and exhibit superior spiritual care competence (43, 44). ③ The hospital ethical climate can also indirectly affect spiritual care competence through professional identity assessment. This may stem from the fact that, guided by the hospital ethical climate, nurses can enhance their sense of value identification, psychological belonging, and work enthusiasm toward the hospital, thereby bolstering their professional identity (45, 46). Consequently, managers should acknowledge the impact of the hospital ethical climate on nurses' spiritual care competence, foster a conducive organizational ethical climate, and guide nurses in establishing correct ethical principles. Additionally, enriching team-building activities outside of work can foster camaraderie, create a harmonious departmental atmosphere, and provide nurses with greater respect and support.

### 4.5 Limitation

Due to constraints related to time, manpower, and resources, this study was confined to a single tertiary hospital, potentially compromising the representativeness of the sample.

## 5 Conclusions

In summary, this study conducted a thorough analysis of the multidimensional influencing factors and their mechanisms regarding the spiritual care competence of orthopedic nurses. This exploration is grounded in a robust theoretical framework, specifically the Person-Environment Fit theory and the Triadic Reciprocal Determinism theory. The findings reveal that the hospital's ethical climate can directly and positively impact spiritual care competence, while also indirectly influencing it through spiritual care perspectives and professional identity assessments. For future research, it is recommended to broaden the sample size to encompass a wider range of medical institutions across different regions, as well as orthopedic nurses with diverse characteristics. Additionally, longitudinal designs or experimental intervention methods should be employed to further elucidate the causal relationships between various influencing variables and spiritual care competence within the current structural equation model. This will facilitate the development of a more comprehensive framework for enhancing spiritual care competence, thereby providing clearer evidence to support relevant theoretical research and interventions.

## Data Availability

The raw data supporting the conclusions of this article will be made available by the authors, without undue reservation.

## References

[1] Nissen RD Viftrup DT Hvidt NC. The process of spiritual care. Front Psychol. (2021) 7:674453. 10.3389/fpsyg.2021.674453 34557128 PMC8453153

[2] Ramezani M Ahmadi F Mohammadi E Kazemnejad A. Spiritual care in nursing: a concept analysis. Int Nurs Rev. (2014) 61:211–9. 10.1111/inr.12099 24712404

[3] Niu Y McSherry W Partridge M. Exploring the meaning of spirituality and spiritual care in Chinese contexts: a scoping review. J Relig Health. (2022) 61:2643–62. 10.1007/s10943-021-01199-5 33624216

[4] Heidari A Afzoon Z Heidari M. The correlation between spiritual care competence and spiritual health among Iranian nurses. BMC Nurs. (2022) 21:277. 10.1186/s12912-022-01056-0 36224620 PMC9555262

[5] Fang HF Susanti HD Dlamini LP Miao NF Chung MH. Validity and reliability of the spiritual care competency scale for oncology nurses in Taiwan. BMC Palliat Care. (2022) 21:16. 10.1186/s12904-022-00903-w 35114991 PMC8815162

[6] Shi X Wang F Xue L Gan Z Wang Y Wang Q. Current status and influencing factors of spiritual needs of patients with advanced cancer: a cross-sectional study. BMC Nurs. (2023) 22:131. 10.1186/s12912-023-01306-9 37076918 PMC10116731

[7] Amiri M Mirzaei S Nasiriani K. Effect of spiritual care on anxiety and fear of orthopaedic surgery Patients. J Pastoral Care Counsel. (2021) 75:259–66. 10.1177/15423050211055390 34851205

[8] Costeira C Querido A Ventura F Loureiro H Coelho J Benito E. Spiritual care[givers] competence in palliative care: a scoping review. Healthcare. (2024) 12:1059. 10.3390/healthcare12111059 38891134 PMC11171750

[9] Edgley C Hogg M De Silva A Braat S Bucknill A Leslie K. Severe acute pain and persistent post-surgical pain in orthopaedic trauma patients: a cohort study. Br J Anaesth. (2019) 123:350–9. 10.1016/j.bja.2019.05.030 31248645

[10] Xu Y Liu K Chen K Feng M. How does person-environment fit relate to career calling? The role of psychological contracts and organizational career management. Psychol Res Behav Manag. (2023) 16:1597–614. 10.2147/PRBM.S404374 37159647 PMC10163889

[11] Yin M Zhang W Evans R Zhu C Wang L Song J. Violence on the front line: a qualitative comparative analysis of the causes of patient violence towards medical staff in China during the COVID-19 pandemic. Curr Psychol. (2023) 2:1–21. 10.1007/s12144-023-04456-w 37359625 PMC9979127

[12] Jiang W Zhao X Jiang J Zhang H Sun S Li X. The association between perceived hospital ethical climate and self-evaluated care quality for COVID-19 patients: the mediating role of ethical sensitivity among Chinese anti-pandemic nurses. BMC Med Ethics. (2021) 22:144. 10.1186/s12910-021-00713-4 34706723 PMC8549414

[13] Noh YG Kim SY. Factors of Hospital Ethical Climate among Hospital Nurses in Korea: a systematic review and meta-analysis. Healthcare. (2024) 12:372. 10.3390/healthcare12030372 38338257 PMC10855336

[14] Fradelos EC Tzavella F. Spiritual climate as is perceived by Greek clinical nurses. A validation study. Mater Sociomed. (2020) 32:66–70. 10.5455/msm.2020.32.66-70 32410895 PMC7219727

[15] Nie YG Li JB Dou K Situ QM. The associations between self-consciousness and internalizing/externalizing problems among Chinese adolescents. J Adolesc. (2014) 37:505–14. 10.1016/j.adolescence.2014.04.002 24931553

[16] Ren Y. Research on the Current Situation and Countermeasures of Professional Self Development of Ethnic Minority Teachers in Xinjiang. Xinjiang Normal University (2011).

[17] Garza B Bang SH Lin LC. Professional self-concept of BSN students: a cross-sectional correlational study. Nurse Educ Today. (2024) 139:106238. 10.1016/j.nedt.2024.106238 38728990

[18] Chang T Jiang X Wei J Zhao J Li Z Li H. Mediating effects of psychological capital on the relationship between workplace violence and professional identity among nurses working in Chinese public psychiatric hospitals: a cross-sectional study. BMJ Open. (2023) 13:e065037. 10.1136/bmjopen-2022-065037 36599638 PMC9815003

[19] Zhu J Xie X Pu L Zou L Yuan S Wei L. Relationships between professional identity, motivation, and innovative ability among nursing intern students: a cross-sectional study. Heliyon. (2024) 10:e28515. 10.1016/j.heliyon.2024.e28515 38596131 PMC11002581

[20] Yüksel Kaçan C. Does the nursing students' perceived level of compassion correlate with affect their perception of spiritual care? Nurse Educ Today. (2023) 130:105951. 10.1016/j.nedt.2023.105951 37657256

[21] Taylor EJ Highfield M Amenta M. Attitudes and beliefs regarding spiritual care. A survey of cancer nurses. Cancer Nurs. (1994) 17:479–87. 10.1097/00002820-199412000-00005 7820826

[22] Wei D Liu X Chen Y Zhang M Mao T Fu Y. Reliability and validity of Chinese Version of Spiritual Care Competence scale. Chin Nurs Manag. (2017) 17:1610–5. 10.3969/j.issn.1672-1756.2017.12.008

[23] Liu D Zhan Y Wang M Fang C. Reliability and validity of Chinese version of the Spiritual Care Perspective Scale. Chin Nurs Res. (2021) 35:3761–6. 10.12102/j.issn.1009-6493.2021.21.00135585279

[24] Liu L Hao Y Liu X. Development of professional identity scale for nurses. Nurs J Chin PLA. (2011) 28:18–20. 10.3969/j.issn.1008-9993.2011.03.006

[25] Olson LL. Hospital nurses' perceptions of the ethical climate of their work setting. Image J Nurs Sch. (1998) 30:345–9. 10.1111/j.1547-5069.1998.tb01331.x 9866295

[26] Wang L. Reliability and Validity of Chinese Version of the Hospital Ethical Climate Survey Assessment and the application in Nurses. Zhengzhou University (2018).

[27] Li L Lv J Zhang L Song Y Zhou Y Liu J. Association between attitude towards death and spiritual care competence of Chinese oncology nurses: a cross-sectional study. BMC Palliat Care. (2021) 20:150. 10.1186/s12904-021-00846-8 34587921 PMC8480268

[28] Cao Y Kunaviktikul W Petrini M Sripusanapan A. Proposing a conceptual framework of spiritual care competence for Chinese nurses. Nurs Health Sci. (2020) 22:498–506. 10.1111/nhs.12692 32104965

[29] Hu Y Li F Chiou JF. Psychometric properties of the Chinese mainland version of the Palliative Care Spiritual Care Competency Scale (PCSCCS-M) in nursing: a cross-sectional study. BMC Palliat Care. (2019) 18:27. 10.1186/s12904-019-0409-6 30849968 PMC6408799

[30] O'Brien MR Kinloch K Groves KE Jack BA. Meeting patients' spiritual needs during end-of-life care: a qualitative study of nurses' and healthcare professionals' perceptions of spiritual care training. J Clin Nurs. (2019) 28:182–9. 10.1111/jocn.14648 30091251

[31] Cheng Q Liu X Li X Wang Y Lin Q Qing L. Spiritual care competence and its relationship with self-efficacy: AN online survey among nurses in mainland China. J Nurs Manag. (2021) 29:326–32. 10.1111/jonm.13157 32914508

[32] Sobanski PZ Alt-Epping B Currow DC Goodlin SJ Grodzicki T Hogg K. Palliative care for people living with heart failure: European Association for Palliative Care Task Force expert position statement. Cardiovasc Res. (2020) 116:12–27. 10.1093/cvr/cvz200 31386104

[33] Liu Q Ho KY Lam KK Lam WY Cheng EH Ching SS. A descriptive and phenomenological exploration of the spiritual needs of Chinese Children Hospitalized with Cancer. Int J Environ Res Public Health. (2022) 19:13217. 10.3390/ijerph192013217 36293795 PMC9602965

[34] Hoosen M Roman NV Mthembu TG Naseer M. Unani Tibb practitioners' perceptions and attitudes towards spirituality and spiritual care in Unani Tibb practice in South Africa. BMC Comp Med Ther. (2023) 23:189. 10.1186/s12906-023-04002-y 37296450 PMC10251630

[35] Elliott R Wattis J Chirema K Brooks J. Mental health nurses' understandings and experiences of providing care for the spiritual needs of service users: a qualitative study. J Psychiatr Ment Health Nurs. (2020) 27:162–71. 10.1111/jpm.12560 31495046

[36] Mthembu TG Roman NV Wegner L. A cross-sectional descriptive study of occupational therapy students' perceptions and attitudes towards spirituality and spiritual care in occupational therapy education. J Relig Health. (2016) 55:1529–45. 10.1007/s10943-015-0125-3 26374135

[37] Wang H Yang M. Influence of professional identity on the e-learning adaptability among Chinese Nursing Students During COVID-19. Front Public Health. (2022) 9:754895. 10.3389/fpubh.2021.754895 35155333 PMC8829332

[38] Hojat M Badiyepeymaiejahromi Z. Relationship between Spiritual Intelligence and Professional Self-concept among Iranian Nurses. Invest Educ Enferm. (2021) 39:e12. 10.17533/udea.iee.v39n3e12 34822239 PMC8912165

[39] Okumoto A Yoneyama S Miyata C Kinoshita A. The relationship between hospital ethical climate and continuing education in nursing ethics. PLoS ONE. (2022) 17:e0269034. 10.1371/journal.pone.0269034 35862376 PMC9302802

[40] Yang D Liu Y Zhang H Zhang Y. The effect of family boundary flexibility on employees' work engagement: a study based on person-environment fit theory perspective. Front Psychol. (2023) 14:1185239. 10.3389/fpsyg.2023.1185239 37842711 PMC10568136

[41] Schluter J Winch S Holzhauser K Henderson A. Nurses' moral sensitivity and hospital ethical climate: a literature review. Nurs Ethics. (2008) 15:304–21. 10.1177/0969733007088357 18388166

[42] Cai H Zhu L Jin X. Construed organizational ethical climate and whistleblowing behavior: the moderated mediation effect of person-organization value congruence and ethical leader behavior. Behav Sci. (2024) 14:293. 10.3390/bs14040293 38667088 PMC11047732

[43] Dziurka M Ozdoba P Olson L Jedynak A Ozga D Jurek K. Hospital ethical climate survey - selected psychometric properties of the scale and results among polish nurses and midwives. BMC Nurs. (2022) 21:295. 10.1186/s12912-022-01067-x 36324181 PMC9628138

[44] Zhang N Xu D Bu X Xu Z. Latent profiles of ethical climate and nurses' service behavior. Nurs Ethics. (2023) 30:626–41. 10.1177/09697330231160008 36935448

[45] Barattucci M Teresi M Pietroni D Iacobucci S Lo Presti A Pagliaro S. Ethical climate(s), distributed leadership, and work outcomes: the mediating role of organizational identification. Front Psychol. (2021) 11:564112. 10.3389/fpsyg.2020.564112 33613349 PMC7889511

[46] Teresi M Pietroni DD Barattucci M Giannella VA Pagliaro S. Ethical climate(s), organizational identification, and employees' behavior. Front Psychol. (2019) 10:1356. 10.3389/fpsyg.2019.01356 31275196 PMC6593040

